# Predictors of Functional Outcome After Microsurgical Resection of Brain Arteriovenous Malformations: A Retrospective Single-Center Study

**DOI:** 10.3390/jcm14248680

**Published:** 2025-12-08

**Authors:** Aleksandar Janicijevic, Jelena Kostic, Nikola Jovicevic, Aleksandar Milosavljevic, Dean Vidovic, Milica Cancarevic-Janicijevic, Nikola Repac, Goran Tasic

**Affiliations:** 1 Clinic of Neurosurgery, University Clinical Center of Serbia, 11000 Belgrade, Serbia; aleksandarjanicijevic82@gmail.com (A.J.); n.jovicevic05@gmail.com (N.J.); angie3bg@yahoo.com (G.T.); 2 Faculty of Medicine, University of Belgrade, 11000 Belgrade, Serbia; 3 Clinic of Neurosurgery, University Clinical Center of Kragujevac, 10486 Kragujevac, Serbia; 4 Faculty of Medical Sciences, University of Kragujevac, 34000 Kragujevac, Serbia; 5 General Hospital Doboj, 74000 Doboj, Bosnia and Herzegovina; 6 Special Hospital for Cerebrovascular Diseases “Sveti Sava”, 11000 Belgrade, Serbia

**Keywords:** brain arteriovenous malformation, microsurgery, mRS score, functional outcome

## Abstract

**Background/Objectives:** Brain arteriovenous malformations (AVMs) are rare but clinically significant lesions associated with hemorrhage, seizures, and neurological deficits. Microsurgical resection remains the gold standard for low- and intermediate-grade AVMs, though treatment of unruptured AVMs is still debated. To present functional outcomes of microsurgical treatment for intracranial AVMs in a high-volume neurosurgical center. **Methods:** We retrospectively analyzed 111 patients who underwent microsurgical AVM resection between 2010 and 2022 at the Clinical Center of Serbia. Demographic, clinical, radiological, and surgical data were collected. Functional outcomes were assessed using the modified Rankin Scale (mRS) at discharge and after nine months. **Results:** The mean patient age was 36 years (range 8–75); 54 (48.6%) were male. AVMs were most often supratentorial (91.9%), located in the frontal (26.1%) and temporal lobes (19.8%). Hemorrhage was the presenting symptom in 53.2% of patients. Postoperative complications included hematoma (10.8%), meningitis (13.5%), and wound infection (8.1%). The mean mRS at discharge was 2.10, improving significantly to 1.15 at nine months (*p* < 0.001). Favorable outcome (mRS ≤ 2) was achieved in 64.0% at discharge and 81.1% at nine months. Prognostic factors for poor outcome included AVM size larger than 6 cm, higher supplementary Spetzler–Martin grade, and combined venous drainage. **Conclusions:** Microsurgical resection provides an important component of multidisciplinary AVM management, especially in low- and selected intermediate-grade lesions, achieving favorable functional outcomes in the majority of patients. Careful patient selection, AVM grading, and venous drainage analysis remain essential for prognosis and treatment planning.

## 1. Introduction

Brain arteriovenous malformations (bAVMs) are defined as complex cerebrovascular anomalies characterized by direct arteriovenous communications without an intervening capillary network. AVMs are uncommon, affecting 10–18 per 100,000 adults, with an incidence of 1 case per 100,000 annually, but they cause about 2% of all hemorrhagic strokes [1]. Clinical presentations vary and may include intracranial hemorrhage, seizures, focal neurological deficits, or nonspecific headaches; many cases are incidentally found on MRI.

The natural history of AVMs carries a significant risk of rupture, which is the primary reason for diagnostic workup and therapeutic planning.

The annual risk of bleeding from unruptured AVMs (ubAVMs) ranges from 2.1% to 4.12%, and over 20 years, the cumulative risk is estimated to be about 29% [2,3].

Hemorrhagic events can result in substantial morbidity and mortality. Reported mortality rates range from 12% to 66.7%, while permanent neurological deficits are observed in 23% to 40% of patients [4,5].

Assessment of rupture risk and planning of treatment require a comprehensive, multiparametric approach. Key factors include nidus size, anatomical location, number and configuration of feeding arteries, and the pattern of venous drainage. These elements are essential for assessing surgical accessibility and selecting the appropriate therapeutic modality. Additional angioarchitectural features—such as venous ectasia or intranidal aneurysms—may further increase rupture risk and influence procedural strategy [3].

Epidemiological data suggest a slight male predominance among AVM patients. Cases occur across a broad age range, from childhood to late adulthood. No significant sex-based differences have been found in AVM location (supratentorial vs. infratentorial) or hemispheric distribution. However, anatomical localization may still have prognostic and therapeutic implications [4].

While there is broad consensus that ruptured arteriovenous malformations (rAVMs) require complete obliteration or surgical excision, the optimal management of unruptured AVMs remains a subject of ongoing debate. This discussion has intensified in the wake of the ARUBA trial (A Randomized Trial of Unruptured Brain Arteriovenous Malformations), which showed that medical management alone was superior to interventional therapy for the prevention of death or stroke in patients with unruptured AVMs followed for 33 months [6]. However, the ARUBA trial has several important limitations. Only 226 out of 1740 eligible patients (13%) were randomized, raising concerns about selection bias. Several authors criticized the low number of patients treated with microsurgery alone (15.8%) in the ARUBA trial. They pointed out that embolization and stereotactic radiosurgery—both known for lower rates of complete AVM obliteration—were the dominant treatment methods. This likely influenced the results and may have led to the incorrect impression that microsurgery is ineffective [7,8,9,10].

Microsurgery is still considered the gold standard for low-grade arteriovenous malformations (AVMs), as classified by currently established grading systems, including the Spetzler–Martin, Spetzler–Ponce and Supplementary Spetzler–Martin [11,12,13]. It offers high obliteration rates and favorable long-term outcomes [5].

This study presents our institutional experience with microsurgical treatment of brain arteriovenous malformations (AVMs). We analyzed clinical presentation, angioarchitectural characteristics and surgical outcomes. Functional status was assessed both at discharge and at 9-month follow-up. Additionally, we examined factors associated with ruptured AVMs and functional recovery. These findings may help improve selection criteria and support individualized treatment planning for AVM patients.

## 2. Materials and Methods

This retrospective, single-center study included patients with angiographically confirmed brain arteriovenous malformations (bAVMs) who were treated at the Cerebrovascular Department of the Clinic for Neurosurgery, University Clinical Center of Serbia, over a twelve-year period between January 2010 and January 2022.

All cases were assessed by a multidisciplinary neurovascular board. The board consisted of a vascular neurosurgeon, an interventional neuroradiologist and a radiation oncologist. Treatment decisions were made by consensus. The selection process incorporated clinical presentation, lesion morphology, angioarchitectural features, and individual patient factors.

Available treatment modalities included conservative observation, microsurgical resection, stereotactic radiosurgery (SRS), endovascular embolization, and various multimodal combinations. Each therapeutic strategy was chosen individually, with the aim of optimizing treatment efficacy while minimizing procedural risk. In our study, we included only patients with brain AVMs who underwent primary microsurgical treatment during the study period. Both ruptured and unruptured AVMs were included. Patients with any previous intervention (embolization, combined surgery, radiosurgery or prior surgery) were excluded, ensuring a uniform cohort of previously untreated lesions. At our institution, microsurgery is the preferred treatment in cases where the AVM is deemed microsurgical accessible. Treatment allocation was based on a combination of anatomical factors, patient age, clinical condition, and multidisciplinary consensus. In general, bAVMs are classified with Supplementary Spetzler–Martin (Suppl. SM) grades of ≤6 and were primarily treated with microsurgical resection. This approach was considered the most effective and definitive option for low-grade lesions.

For higher-grade AVMs, treatment selection was individualized. Decisions were based on multiple factors, including clinical status of patients, nidus size, anatomical location, compactness or diffuseness of the lesion, and the eloquence of adjacent brain tissue. The decision to proceed with microsurgery in high-grade cases was further influenced by the surgical team’s experience and intraoperative assessment of the lesion’s accessibility.

Preoperative evaluation included detailed analysis of the nidus position, proximity to eloquent structures, and anticipated surgical complexity. These elements were carefully considered to ensure that resection was both feasible and safe.

Lesions graded as Suppl. SM 9 or 10 were generally managed conservatively. This approach was especially favored in elderly patients and in those with significant comorbidities, where the risks of intervention were judged to outweigh the potential therapeutic benefits. Exclusion criteria included patients with other congenital vascular lesions such as vein of Galen malformations, dural arteriovenous fistulas, cavernous malformations, and venous malformations. Also, patients who underwent radiosurgical or endovascular treatment at any given time were not included in this study.

Collected data also included demographic characteristics, AVM characteristics including localization, size, arterial feeder origin and number, venous drainage patterns, number of drainage veins, presence of venous ectasia and clinical presentation. Neurological assessment was performed in the immediate postoperative period until discharge, and then after nine months.

All bAVMs were classified using the supplemented Spetzler–Martin (Supp-SM) grading system and divided into two groups: Supp-SM ≤ 6 (low-risk group) and Supp-SM > 6 (high-risk group). Functional status was assessed using the modified Rankin Scale (mRS) in the immediate postoperative period (until hospital discharge) and at the 9-month follow-up. Based on mRS scores, patients were categorized into two outcome groups: mRS ≤ 2 (good outcome) and mRS > 2 (poor outcome).

Postoperative outcomes were assessed using standardized clinical and radiological follow-up. Functional status was evaluated with the modified Rankin Scale (mRS) at discharge and again at the 9-month follow-up. Early postoperative complications, including new hemorrhage or hematoma detected on CT, were systematically documented. Permanent neurological morbidity was not assessed separately, as the study focused primarily on mRS-based functional outcomes.

Radiological confirmation of complete AVM obliteration was recorded only for patients who underwent follow-up DSA or CTA imaging, acknowledging that angiographic data were not available for the entire cohort.

Statistical analysis was performed using the Statistical Package for the Social Sciences (SPSS Inc., Chicago, IL, USA), version 25.0. Demographic data and AVM characteristics were summarized using descriptive statistics for continuous variables (mean ± standard deviation) and categorical variables (number and percentage). *t*-tests were used for numerical data, and Fisher’s exact test for categorical data. Statistical significance was set at *p* < 0.05.

## 3. Results

Table 1 presents the patient demographics and AVM anatomical characteristics stratified by grade (Low grade/High grade).

The study included 111 patients, 53 (47.7%) male and 58 (52.3%) female. There was no statistically significant difference between the number of male and female patients.

Patients with ruptured (N = 59) and unruptured AVMs (N = 52) showed similar baseline characteristics (Table 1). Median age was comparable between groups (36.6 vs. 38.8 years). Gender distribution was balanced, with slightly more females in the ruptured group (55.9%) and more males in the unruptured group (51.9%).

Medium-sized AVMs (3–6 cm) were most common in both cohorts (59.3% ruptured vs. 54.4% unruptured). Laterality was evenly distributed (left-sided: 52.5% vs. 51.9%).

Regarding localization, ruptured AVMs were most frequently frontal (20.3%), temporal (16.9%), occipital (13.6%), and cerebellar (13.6%). Unruptured AVMs were most often frontal (28.5%), parietal (17.3%), and motor cortex lesions (15.4%). Ventricular lesions occurred only in unruptured AVMs, while brainstem AVMs appeared only in the ruptured group.

The mean age of patients with low-grade AVMs (N = 93) was 36.5 years, while those with high-grade AVMs (N = 18) presented a higher mean age of 43.2 years. Males constituted 47.7% of the total cohort, with a predominance in the high-grade group (77.8%) compared to 41.9% in the low-grade group.

Among all 111 patients, 35 (31.5%) had small AVMs, 69 (62.2%) had medium AVMs, and 7 (6.3%) had large AVMs. In the low-risk group (N = 93), 35 patients (37.6%) had small AVMs, 54 (58.1%) had medium AVMs, and 4 (4.3%) had large AVMs. In the high-risk group (N = 18), no patients had small AVMs, 15 (83.3%) had medium AVMs, and 3 (16.7%) had large AVMs.

With respect to localization, AVMs were left-sided in 52.3% of cases and right-sided in 47.7%. In low-grade AVMs, the most frequent locations were the frontal (29%), parietal (16.1%), temporal (15.1%), and occipital (15.1%) lobes. Conversely, high-grade AVMs were more often situated in functionally eloquent areas, particularly within the motor cortex (44.4%) and deep brain structures (22.2%). Occipital and cerebellar AVMs were rare in the high-grade group. Ventricular and brainstem locations were uncommon, each representing a small proportion of the overall cohort (Table 1).

Venous ectasia was identified in 60 patients (54.1%). It was more frequently associated with high-grade lesions (72.2%) compared with low-grade lesions (50.5%). Regarding venous drainage patterns, superficial drainage was the most prevalent overall (61.3%), observed predominantly in the low-grade group (72%). Deep venous drainage was present in 18.0% of all cases, while combined superficial and deep drainage was identified in 20.7% of patients. Combined drainage was characteristic of high-grade AVMs, observed in 77.8% of these lesions, in contrast to 9.7% of the low-grade group.

Regarding feeding artery supply, across the entire cohort, the most common arterial feeders were the middle cerebral artery (MCA), present in 55.9% of cases, followed by the anterior cerebral artery (ACA) in 36.0%, and the posterior cerebral artery (PCA) in 25.2% of patients.

When stratified by AVM grade, high-grade lesions demonstrated a predominance of MCA and PCA supply. Specifically, the MCA was involved in 66.7% of high-grade AVMs, while the PCA contributed in 33.3% of cases. In contrast, pericallosal artery feeders were observed exclusively in low-grade AVMs. These feeders were identified in 22.6% of low-grade cases and were absent in all high-grade lesions (0%).

ACA supply was relatively common in both subgroups, with a comparable distribution between low-grade (36.6%) and high-grade (33.3%) AVMs.

Arterial supply from smaller posterior circulation vessels, such as the superior cerebellar artery (SCA) and the posterior inferior cerebellar artery (PICA), as well as from branches of the internal carotid artery (ICA) and posterior communicating artery (PCoA), was infrequent across the entire cohort. Due to the small number of such cases, no consistent or grade-specific pattern could be identified for these arterial sources (Table 2).

An AVM rupture was the most frequent mode of clinical onset, occurring in 59 patients (53.2%) overall. Rupture was observed in 52.7% of the low-grade group and in 55.6% of the high-grade group. Non-ruptured presentations accounted for 46.8% of all cases. Among the non-hemorrhagic presentations, epileptic seizures and focal neurological symptoms were common. Generalized seizures occurred in 34.2% of patients, including 35.5% in the low-grade and 27.8% in the high-grade group. Partial epileptic seizures were observed in 12.6% of the total cohort, more frequently among high-grade AVMs (22.2%) than low-grade lesions (10.8%). Neurological deficits were recorded in 37 patients (33.3%), being more prevalent in the high-grade category (50.0%) than in the low-grade one (30.1%). Headache was another frequent presenting symptom, reported by 51.4% of patients overall, including 45.2% in the low-grade and 66.7% in the high-grade group (Table 3).

At the initial assessment on discharge, good outcomes were achieved in 82.7% of low-grade patients (26 ruptured and 41 non-ruptured) compared with only 26.3% of high-risk cases (1 ruptured and 3 non-ruptured). The proportion of poor outcomes at discharge increased progressively with AVM risk level, reaching 90.0% in high-risk ruptured lesions (*p* = 0.001 for low vs. high risk). At nine-month follow-up, outcomes improved across all categories. Good outcomes were observed in 90.5% of low-risk patients and 69.6% of high-risk patients. Despite improvement in both groups, high-risk AVMs continued to show a lower proportion of favorable results (*p* = 0.04). The greatest functional recovery was observed among high-risk ruptured AVMs, where the proportion of good outcomes increased from 10.0% to 66.7% between the two assessments.

Patients with superficial venous drainage (N = 65; 58.6%) showed predominantly favorable outcomes (good: 50 at discharge; 58 at 9 months), with a statistically significant association (*p* = 0.03).

Those with deep drainage (N = 22; 19.8%) had an equal distribution of good and poor outcomes at discharge (11 vs. 11), improving to 17 good and 2 poor outcomes at 9 months.

Patients with combined drainage (N = 24; 21.6%) showed more balanced distributions (good: 10 at discharge; 16 at 9 months; poor: 14 and 4, respectively) (Table 4).

Supplementary Spetzler–Martin score was significantly associated with hemorrhagic presentation, with an OR of 2.429 (95% CI: 1.178–5.011; *p* = 0.016). This suggests that higher grading in this parameter markedly increases the likelihood of hemorrhage, acting as a strong predictor.

Superficial venous was significantly protective against hemorrhagic presentation (OR = 0.111; 95% CI: 0.017–0.709; *p* = 0.020), indicating that superficial AVMs are less prone to hemorrhage.

Other factors, including AVM size, side of AVM, number of feeders and drainage veins as well as deep draining veins and presence of venous ectasia, showed no statistically significant association with hemorrhagic presentation (*p* > 0.05) (Table 5).

The multivariable logistic regression analysis revealed that mean ranking score after 9 months was significantly associated with supplementary Martin–Spetzler grade (*p* < 0.001) and size of AVM (*p* = 0.025). Specifically, higher S&M grades were linked to increased odds of lower mRank scores, with an odds ratio (OR) of 1.636 (95% CI: 1.265 to 2.115). Larger AVM size categories were associated with decreased odds of lower mRS at 9 months (OR = 0.647, 95% CI: 0.453 to 0.925).

Additional variables such as the presence of venous drainage, arterial supply, initial presentation, and IVH showed varying degrees of association, but their confidence intervals encompassed unity, indicating no statistically significant effect in this model. Notably, the collinearity diagnostics suggested low multicollinearity among the predictors, with VIF values below 5.

Based on the initial hemorrhage, increased initial presentation severity, represented by higher values or categories, is associated with poorer outcomes in mRank scores, with an odds ratio (OR) of approximately 1.769 (95% CI: 0.072 to 1.070). Despite the confidence interval crossing below 1, the *p*-value of 0.026 indicates a statistically significant association. This suggests that patients presenting with hemorrhage tend to have worse mean ranking scores at 9 months, emphasizing the importance of initial hemorrhagic presentation as a predictor (Table 6).

At discharge, 42 patients (37.8%) had an mRS score of 1, all belonging to the low-risk group (45.2%), while no high-risk patients achieved this score. An mRS score of 2 was recorded in 29 patients (26.1%), including 25 (26.9%) in the low-risk and 4 (22.2%) in the high-risk group. Scores of 3 and 4 were observed in 11 (9.9%) and 17 (15.3%) patients, respectively, with higher proportions in the high-risk group (22.2% each) compared with the low-risk group (7.5% and 14.0%). Only one patient (0.9%) had an mRS of 5, belonging to the high-risk group (5.6%), while 11 patients (9.9%) had an mRS of 6, including 6 (6.5%) in the low-risk and 5 (27.8%) in the high-risk group.

At 9-month follow-up, functional improvement was observed across both groups. A total of 26 patients (23.4%) achieved an mRS of 0, all in the low-risk group (28.0%). An mRS of 1 was recorded in 49 patients (44.1%), including 46 (49.5%) in the low-risk and 3 (16.7%) in the high-risk group. Fifteen patients (13.5%) had an mRS of 2, 9 (9.7%) low-risk and 6 (33.3%) high-risk. Higher mRS scores of 3 and 4 were noted in 9 (8.1%) and 1 (0.9%) patients, respectively, with the majority belonging to the high-risk subgroup (Table 7).

Intracerebral hemorrhage occurred in 12 patients (10.8%), including 9 (9.7%) in the low-grade group and 3 (16.7%) in the high-grade group (*p* = 0.382). Meningitis was observed in 15 patients (13.5%), with a similar distribution between low-grade (12.9%) and high-grade (16.7%) groups (*p* = 0.708). Wound infection was present in 9 cases (8.1%), 7 (7.5%) in the low-grade and 2 (11.1%) in the high-grade group (*p* = 0.610) (Table 8).

Correlation analysis demonstrated significant association between postoperative intracerebral hemorrhage and meningitis rate, showing a strong positive correlation (*p* = 0.000, r = 0.796), suggesting that patients with postoperative hemorrhage were more likely to develop meningitis. Intracerebral hemorrhage is linked with higher mRS scores at discharge and at follow-up. Patients who developed meningitis also had poorer functional outcomes (r = 0.253, *p* = 0007) (Table 9).

Regarding reoperations, two patients in our cohort required surgical reintervention due to postoperative hematoma. All other postoperative hemorrhages detected on CT were small intraparenchymal or residual hematomas managed conservatively.

At discharge, a good functional outcome (mRS ≤ 2) was achieved in 71 patients (64%), including 67 (72%) in the low-grade group and 4 (22.2%) in the high-grade group (*p* = 0.0001). Poor outcome (mRS > 2) was observed in 40 patients (36%), 26 (28%) low-grade and 14 (77.8%) high-grade. At 9 months, 90 patients (81.1%) demonstrated good outcome, including 81 (87.1%) low-grade and 9 (50%) high-grade (*p* = 0.007). Poor outcome at 9 months was recorded in 10 patients (9.0%), with 6 (6.5%) in the low-grade and 4 (22.2%) in the high-grade group. Mortality is presented in the next tables (Table 10 and Table 11).

The overall mortality rate among all patients was 9.9% (11 deaths), with a 95% confidence interval (CI) of 4.7–15.2%. When stratified by AVM grade, low-grade AVMs had a mortality rate of 6.45% (6 deaths; 95% CI 1.5–11.4%), and high-grade AVMs had a mortality rate of 27.7% (5 deaths; 95% CI 7.1–48.5%). The difference in mortality between low- and high-grade AVMs was statistically significant (*p* = 0.006).

Regarding rupture status, unruptured AVMs showed a mortality rate of 1.9% (1 death; 95% CI 0–5.6%), while ruptured AVMs had a mortality rate of 16.9% (10 deaths; 95% CI 7.3–26.5%), with this difference also reaching statistical significance (*p* = 0.008).

Further stratification by grade and rupture status revealed that low-grade unruptured AVMs had no deaths (0%; 95% CI 0–6.8%, *p* = 0.018), whereas low-grade ruptured AVMs had a mortality rate of 10.1% (6 deaths; 95% CI 3.6–20.8%). High-grade unruptured AVMs showed a mortality rate of 12.5% (1 death; 95% CI 0–35.2%), with a significant *p*-value of 0.007. High-grade ruptured AVMs exhibited the highest mortality rate at 40.0% (4 deaths; 95% CI 9.6–70.4%).

**Figure 1:**
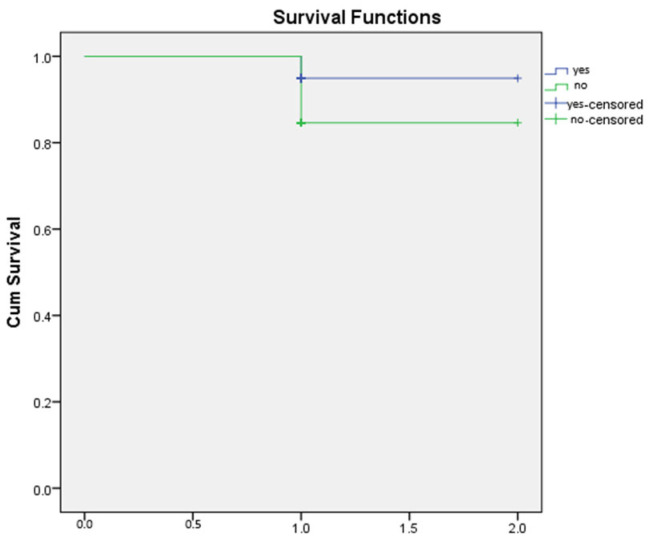
Kaplan-Meier survival curve.

All patients underwent standardized postoperative clinical follow-up with assessment of the modified Rankin Scale (mRS). Postoperative outcomes were evaluated based on the occurrence of rebleeding or new hemorrhage on imaging, and the presence of new, persistent neurological deficits. Radiological confirmation of complete obliteration was recorded for majority of patients, though it is acknowledged that angiographic data were not obtained for the entire cohort (Figure 1).

Kaplan–Meier survival analysis was performed to compare overall survival between patients with and without hemorrhage. As shown in the survival curves, both groups demonstrated high cumulative survival throughout the observation period. The non-hemorrhage group exhibited an initial survival probability of 1.0, with a single decline to approximately 0.87 following one recorded event. Similarly, the hemorrhage group showed a survival drop to approximately 0.90 after its first event. Censored observations in both groups are indicated by “+” markers at the end of individual follow-up times.

**Figure 2:**
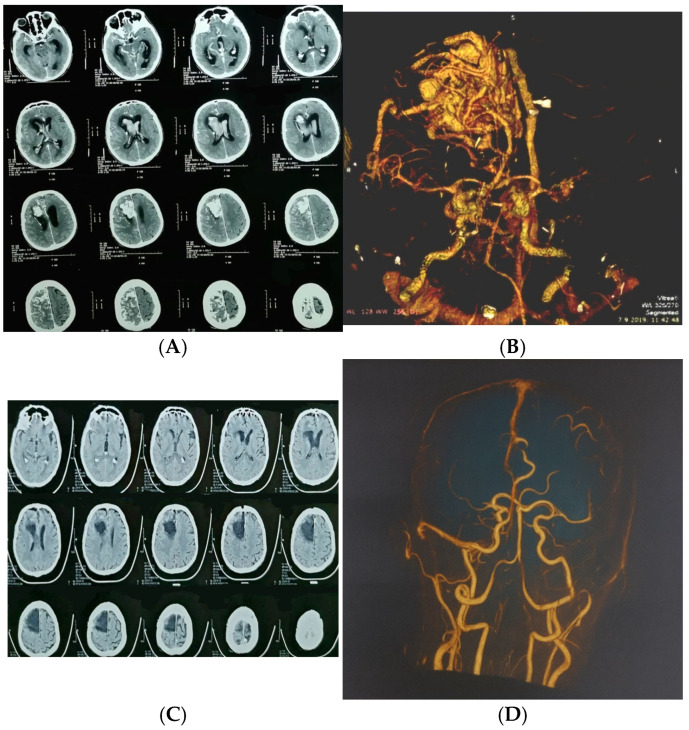
A 65-year-old man presented with severe headache, nausea, vomiting, and impaired consciousness progressing to sopor. (**A**) Axial CT showing large ruptured right frontal arteriovenous malformation (AVM); (**B**) urgent CT angiography confirmed diagnosis; (**C**) postoperative computed tomography; (**D**) postoperative computed tomography angiography.

**Figure 3:**
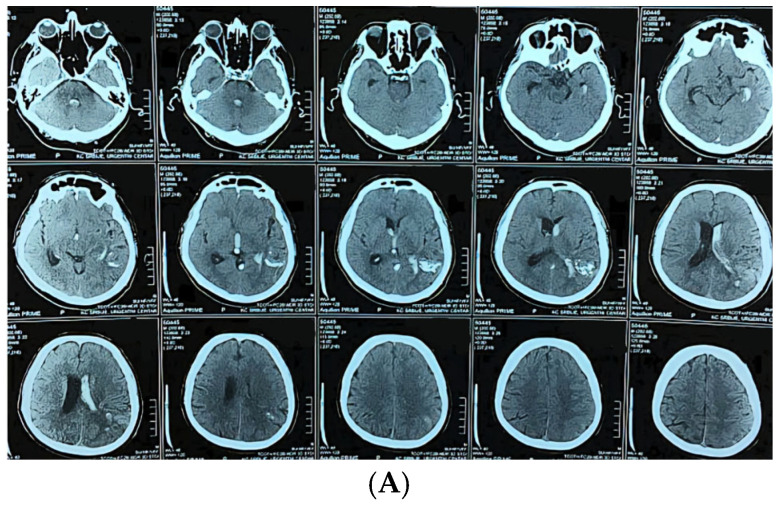

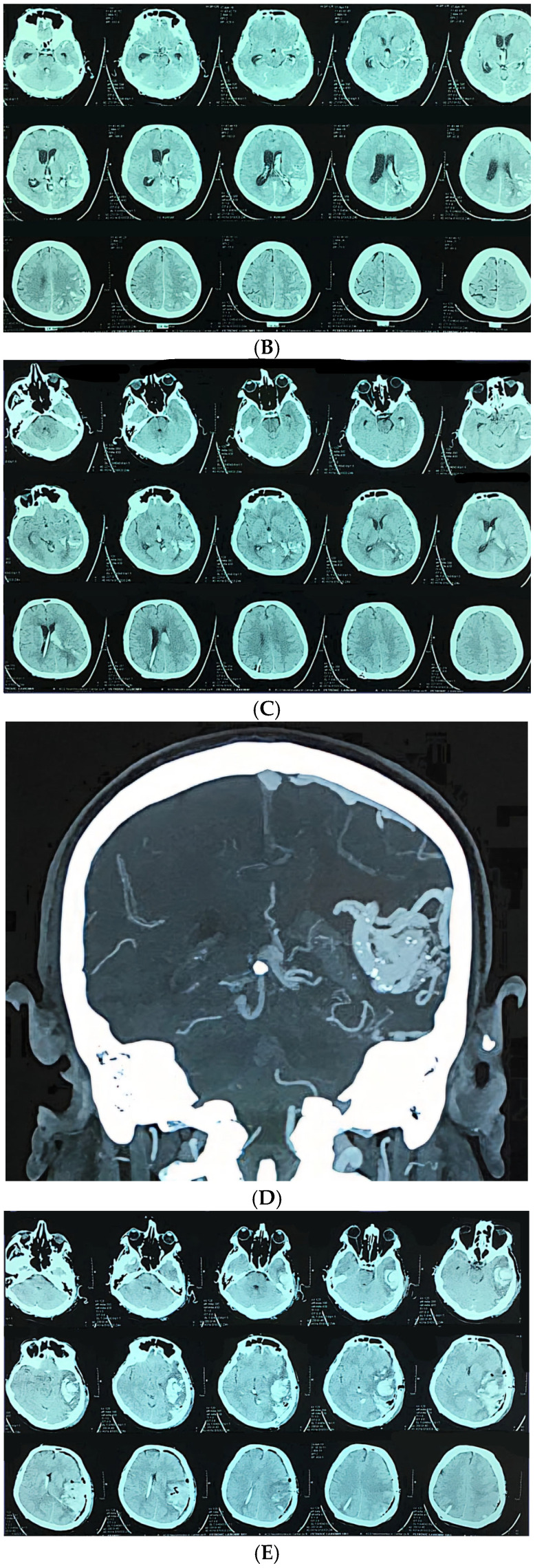

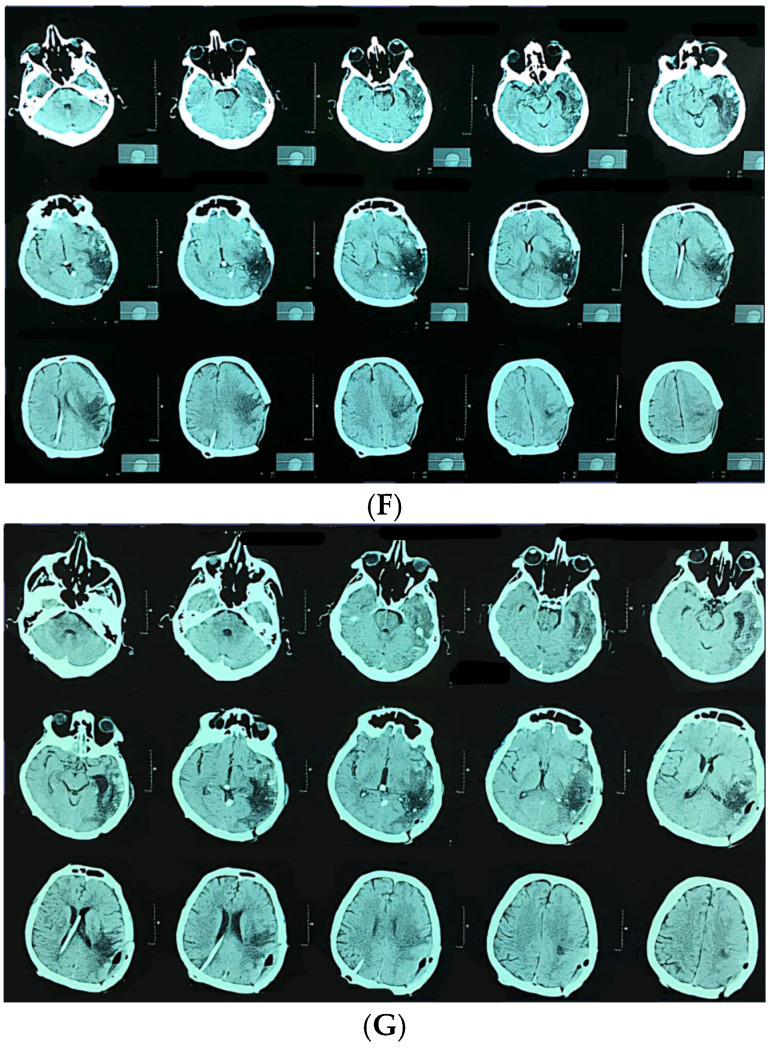
A 58-year-old man presented with sudden onset of headache and vomiting. Brain CT showing initial findings of intraventricular and intracerebral hemorrhage (**A**). Two hours after admission he became unresponsive, GCS 6; urgent CT showed hydrocephalus (**B**). Brain CT after the VP shunt operation (**C**). CT angiography was performed, confirming the diagnosis (**D**). Microsurgical resection of the AVM was performed, and after the operation the patient was unresponsive with hemiplegia. Control brain CT showed cerebral infarction with intracerebral hemorrhage (**E**). CT after the reoperation (**F**) And 6 months after the operation and cranioplasty surgery (**G**).

The survival trajectories of the two groups were largely overlapping, with no substantial divergence across the follow-up interval. This visual similarity suggests that the presence of hemorrhage did not meaningfully affect overall survival within the study period (Figure 2 and Figure 3).

## 4. Discussion

This retrospective cohort study evaluated functional outcomes in patients with brain arteriovenous malformations (AVMs) treated with microsurgical resection. We also analyzed clinical and radiological AVM characteristics.

Our results showed that low-grade AVMs were associated with favorable functional outcomes. Hemorrhage and epilepsy were two most common presenting symptoms. Venous drainage patterns were strongly linked to surgical risk.

Patient demographics in our cohort were consistent with previously published data. This supports the generalizability of our findings. All patients were treated with microsurgical resection. We consider this the gold standard, especially for low-grade AVMs. Our position aligns with previous studies [8,14,15].

None of the patients in our study underwent preoperative embolization. This is consistent with a 2023 meta-analysis that included thirty-two studies. The analysis compared 1088 patients treated with preoperative embolization and 1828 patients treated without it. No statistically significant differences were found between the two groups in terms of AVM obliteration, mortality, complications, poor mRS outcomes, or intraoperative blood loss [16].

In our cohort, all unruptured brain AVMs were stratified as either low-risk (Supp. SM ≤ 6) or high-risk (Supp. SM > 6), based on the Supplementary Spetzler–Martin (Supp. SM) grading system [15]. The threshold score of 6 was selected in line with the findings of Kim et al., who validated this grading system in a large multicenter series of 1009 patients. Their results demonstrated that AVMs with scores ≤6 were associated with acceptable surgical risk and favorable outcomes, while scores above 6 correlated with significantly higher morbidity and mortality [17].

In our cohort of 111 patients, high-grade brain AVMs (Supplementary Spetzler–Martin score > 6) accounted for 16.7% of all cases. The overall mortality rate was 9.9% (11 deaths). Mortality was significantly higher in patients with high-grade AVMs (27.8%) compared to those with low-grade lesions (6.45%) (*p* = 0.006). This finding aligns with the concept that higher Supplementary Spetzler–Martin (Supp. SM) scores reflect more complex angioarchitecture, localization in eloquent areas, and unfavorable venous drainage patterns, all contributing to increased surgical risk and lethality. These results are comparable to the results of Ren Q et al., who reported a postoperative overall mortality rate of 7.9% in a large single-center series of 445 patients. High-grade brain AVMs (Supplementary Spetzler–Martin score > 6) accounted for 17.6% of all their cases [18]. Similarly, Maalim A et al. observed an overall mortality rate of 5.3% in their cohort of 169 surgically treated AVM patients. In their study, high-grade brain AVMs accounted for 15.4% [19]. In contrast, Moon et al. reported a markedly lower mortality rate of 0.4% [8]. However, their cohort differed substantially from ours in case composition: only 5 patients (2.16%) had a Supplementary Spetzler–Martin (Supp. SM) grade of 5, while the remainder had Supp. SM grades below 5. Their series had more low-grade AVMs, which likely resulted in a lower mortality rate than ours, where high-risk lesions made up 16.7%.

These findings further reinforce the clinical relevance and predictive accuracy of the Supplementary Spetzler–Martin grading system, aligning with prior external validation studies [17,20].

Rupture status also had a substantial impact on mortality. Patients presenting with ruptured AVMs had a mortality rate of 16.9%, while those with unruptured lesions showed significantly lower mortality (1.9%) (*p* = 0.008). This is in line with García-Espinosa P. et al. also emphasized hemorrhage as an important factor when it comes to mortality. They reported mortality rate of about 17% in subgroup with ruptured bAVMs [21]. In our study the highest mortality was observed in the subgroup with ruptured high-grade AVMs (40%). Most of these patients presented with severe neurological deficits and underwent urgent, life-saving microsurgical intervention, often as the only viable therapeutic option in the acute setting. In contrast, no deaths were recorded among patients with low-grade, unruptured AVMs.

At discharge, a favorable functional outcome (defined as mRS ≤ 2) was observed in 64.0% of the overall cohort. This proportion was significantly higher among patients with low-grade AVMs (72.0%), compared to only 22.2% in the high-grade group (*p* = 0.0001). By the 9-month follow-up, functional recovery had improved across both risk categories. Good functional outcomes (mRS ≤ 2) were achieved in 81.1% of the entire cohort. The majority of these favorable results were observed in the low-grade group (87.1%). Similarly, Ren Q et al. showed long-term favorable outcomes in 77.3% of all patients. In the low-grade subgroup, they showed favorable outcomes in 84.1% [18]. Also, Theofanis et al. reported favorable outcomes in 228 of 264 patients (86.4%) [22]. In the study of Moon et al., favorable outcomes were achieved in 82.8% of patients [8].

Our findings, together with previously published data, suggest that microsurgical resection can achieve favorable functional outcomes in selected patients with low-grade AVMs, whereas outcomes in high-grade lesions remain substantially less favorable, despite some postoperative improvement.

In the subgroup of high-grade AVMs, only 22.2% of patients achieved favorable functional outcomes (mRS ≤ 2) at discharge. However, by the 9-month follow-up, this proportion increased to 50.0%, indicating a substantial improvement of +27.8%. This suggests that a subset of patients with complex lesions may experience delayed yet meaningful functional recovery. Nevertheless, poor outcomes (mRS > 2) remained common, reflecting the elevated surgical risk and protracted recovery often associated with high-grade AVMs. In the study reported by Li et al., long-term functional improvement occurred in approximately 20% of patients with high-grade AVMs, and favorable functional outcomes (mRS ≤ 2) in 71.4% of patients after extended follow-up [23]. A possible explanation for the difference in outcome rates is the shorter follow-up duration in our study, which was limited to 9 months. Extended observation periods may capture further recovery not evident within early postoperative assessments.

We also conducted a detailed analysis of radiological characteristics and angioarchitectural features of AVMs to identify factors associated with hemorrhagic presentation and surgical risk.

Our results are largely in agreement with those reported by Feghali et al. in the development of the R_2_eD AVM Score, which define risk factors for AVM rupture [24]. In both studies, deep venous drainage was identified as a strong independent predictor of hemorrhagic presentation. In our cohort, deep venous drainage showed an adjusted OR of 2.84 (95% CI: 1.48–5.43; *p* = 0.031), which is comparable to the OR of 2.07 reported in the R_2_eD model. This confirms the role of venous outflow patterns in AVM rupture risk. We also found that deep AVM location was significantly associated with hemorrhage, with an adjusted OR of 2.31 (95% CI: 1.22–4.39; *p* = 0.025). This is almost identical to the OR of 2.3 in the R_2_eD study, supporting the consistency of this anatomical factor across different populations. However, feeding artery characteristics, including the number of feeders and their arterial origin, did not show a significant association with hemorrhagic presentation in our cohort (*p* > 0.05). In contrast, monoarterial supply was a significant factor in the R_2_eD score (OR = 2.24). This discrepancy may be due to differences in patient selection, imaging interpretation, or cohort composition. An additional finding in our study was that left-sided AVMs were more likely to present with hemorrhage (adjusted OR = 1.67; 95% CI: 1.03–2.71; *p* = 0.045). This variable was not evaluated in the R_2_eD model, and its clinical relevance remains uncertain. Further studies are needed to clarify the potential impact of hemispheric laterality on rupture risk. Finally, venous ectasia and the number of draining veins were not associated with hemorrhage in our analysis (*p* > 0.05). These findings are in line with the R_2_eD model, which emphasizes drainage pattern rather than venous morphology.

Interestingly, in our series, AVMs supplied by the pericallosal artery were observed exclusively in the low-grade group (22.6% of low-grade AVMs; 0% in high-grade). Although previous research has not established a direct connection between pericallosal feeders and AVM grade, this observation may be explained by anatomical factors.

The pericallosal artery typically supplies medial aspects of the frontal and parietal lobes, regions often associated with superficial or subcortical AVM location. Lesions in this territory often exhibit simpler angioarchitecture. Specifically, pericallosal-supplied AVMs are less likely to involve multiple arterial feeders, deep venous drainage, or complex nidus morphology—all components that contribute to a higher Spetzler–Martin or Supplementary SM grade. Therefore, the anatomical distribution of the pericallosal artery may underline its exclusive association with low-grade AVMs in our cohort.

The meningitis rate in our cohort (13.5%) is notably higher than the 1–3% reported in most contemporary microsurgical AVM series. This elevated rate appears to be driven primarily by a subgroup of neurologically devastated patients. Eleven patients died postoperatively: one due to sudden cardiac arrest on postoperative day three, and ten others who presented with profound neurological compromise. These patients required prolonged intensive care, often developing hospital-acquired respiratory infections caused by multidrug-resistant organisms, and most underwent external cerebrospinal fluid (CSF) diversion. The combination of critical neurological status, extended ICU stay, nosocomial infections, and the use of external CSF drainage likely contributed to the increased incidence of meningitis observed in this series.

The demographic characteristics of our patients (mean age 36.5 years and equal gender distribution) are comparable to recent series in the literature, which report the most common presentation in the young adult population. The predominant supratentorial localization and the rare occurrence of vertebrobasilar AVMs in our sample are consistent with data from larger cohorts [1].

## 5. Study Limitations

This study has several important limitations. As a retrospective analysis, it is subject to selection and information bias, with limited ability to control for confounding variables. Additionally, the study was conducted at a single tertiary neurosurgical center, which may limit the external validity and generalizability of the findings.

Although the number of patients with hemorrhagic presentation was adequate (59 cases, 53.2%), the proportion of high-grade AVMs was relatively small (N = 18; 16.2%). This reduced the statistical power and precision of estimates in this subgroup, particularly for analyses of functional recovery and mortality.

Another limitation is the relatively short follow-up period of 9 months, which may underestimate long-term functional recovery, especially in patients with severe initial deficits or complex postoperative courses.

Outcome assessment was based solely on the modified Rankin Scale (mRS), which, despite its validity and widespread use, does not capture cognitive or quality-of-life dimensions—factors particularly relevant in younger patients.

This study did not include an endovascular or radiosurgical comparator arm. As a result, direct comparisons of efficacy, safety, or long-term outcomes between microsurgical management and other treatment modalities cannot be made. Our results reflect outcomes in a strictly microsurgical cohort and may not be generalizable to settings where multimodal treatment strategies are routinely employed.

To better understand risk factors and outcomes in AVM patients, future prospective, multicenter studies with extended follow-up and inclusion of additional clinical and patient-reported outcome measures are warranted.

## 6. Conclusions

Despite study limitations, our findings support the role of microsurgery as an important component of multidisciplinary AVM management, especially in low- and selected intermediate-grade lesions. The supplemented Spetzler–Martin (Supp-SM) grading scale proved to be a valid tool for surgical risk stratification, as patients with Supp-SM scores ≤ 6 had significantly better outcomes compared to those with higher scores.

Deep venous drainage and deep-seated location emerged as independent predictors of hemorrhagic presentation, in line with previous models such as the R_2_eD AVM Score. In contrast, the number and arterial origin of feeding vessels were not significantly associated with rupture risk.

Although high-grade AVMs were associated with higher perioperative mortality and worse functional outcomes, a marked improvement in mRS scores over the 9-month follow-up suggests a recovery potential even in complex lesions. Longer follow-up may better illuminate long-term outcomes, especially in patients who presented with life-threatening hemorrhage at admission.

Our findings underscore the importance of an individualized treatment strategy for AVMs, taking into account lesion morphology, functional location, and rupture status at presentation.

## Figures and Tables

**Table 1:** Demographics and AVM anatomical characteristics stratified by grade.

	All Patients	Low Grade	High Grade
	(N = 111)	(N = 93)	(N = 18)
Age (years)	37.60 (8–75)	36.53 (8–75)	43.17 (9–61)
Gender:			
Male	53 (47.7%)	39 (41.9%)	14 (77.8%)
Female	58 (52.3%)	54 (58.1%)	4 (22.2%)
AVM size category:			
<3	35 (31.5%)	35 (37.6%)	0
3–6	69 (62.2%)	54 (58.1%)	15 (83.3%)
>6	7 (6.3%)	4 (4.3%)	3 (16.7%)
Side of AVM:			
Left	58 (52.3%)	49 (52.7%)	9 (50%)
Right	53 (47.7%)	44 (47.3%)	9 (50%)
Superficial venous drainage:			
Yes	68 (61.3%)	67 (72%)	1 (5.6%)
No	43 (38.7%)	26 (28.0%)	17 (94.4%)
Deep venous drainage:			
Yes	20 (18.0%)	17 (18.3%)	3 (16.7%)
No	91 (72%)	76 (81.7%)	15 (83.3%)
Combined venous drainage:			
Yes	23 (20.7%)	9 (9.7%)	14 (77.8%)
No	88 (79.3%)	84 (90.3%)	4 (22.2%)
Presence of venous ectasia:			
Yes	60 (54.1%)	47 (50.5%)	13 (72.2%)
No	51 (55.9%)	46 (49%)	5 (27.8%)
Feeding artery:			
ICA	6 (5.41%)	3 (3.2%)	3 (16.7%)
PCoA	4 (3.6%)	2 (2.2%)	2 (11.1%)
ACA	40 (36.0%)	34 (36.6%)	6 (33.3%)
MCA	62 (55.9%)	50 (53.8%)	12 (66.7%)
Pericallosal	21 (18.9%)	21 (22.6%)	0
PCA	28 (25.2%)	22 (23.7%)	6 (33.3%)
SCA	5 (4.5%)	5 (5.4%)	0
PICA	3 (2.7%)	3 (3.2%)	0
AICA	6 (5.4%)	5 (5.4%)	1 (5.6%)
Localization			
Frontal	27 (24.3%)	27 (29%)	0
Parietal	15 (13.5%)	15 (16.1%)	0
Temporal	17 (15.3%)	14 (15.1%)	3 (16.7%)
Occipital	15 (13.5%)	14 (15.1%)	1 (5.6%)
Motor	14 (12.6%)	6 (6.5%)	8 (44.4%)
Cerebellar	9 (8.1%)	9 (9.7%)	0
Ventricular	4 (3.6%)	4 (4.3%)	0
Deep structures	8 (7.2%)	4 (4.3%)	4 (22.2%)
Brainstem	2 (1.8%)	1 (1.1%)	1 (5.6%)

**Table 2:** Feeding artery supply and venous drainage vein patterns stratified by grade (Low grade/High grade).

	All Patients	Low Grade	High Grade
	(N = 111)	(N = 93)	(N = 18)
Superficial venous drainage			
Yes	68 (61.3%)	67 (72%)	1 (5.6%)
No	43 (38.7%)	26 (28.0%)	17 (94.4%)
Deep venous drainage:			
Yes	20 (18.0%)	17 (18.3%)	3 (16.7%)
No	91 (72%)	76 (81.7%)	15 (83.3%)
Combined venous drainage:			
Yes	23 (20.7%)	9 (9.7%)	14 (77.8%)
No	88 (79.3%)	84 (90.3%)	4 (22.2%)
Presence of venous ectasia:			
Yes	60 (54.1%)	47 (50.5%)	13 (72.2%)
No	51 (55.9%)	46 (49%)	5 (27.8%)
Feeding artery:			
ICA	6 (5.41%)	3 (3.2%)	3 (16.7%)
PCoA	4 (3.6%)	2 (2.2%)	2 (11.1%)
ACA	40 (36.0%)	34 (36.6%)	6 (33.3%)
MCA	62 (55.9%)	50 (53.8%)	12 (66.7%)
Pericallosal	21 (18.9%)	21 (22.6%)	0
PCA	28 (25.2%)	22 (23.7%)	6 (33.3%)
SCA	5 (4.5%)	5 (5.4%)	0
PICA	3 (2.7%)	3 (3.2%)	0
AICA	6 (5.4%)	5 (5.4%)	1 (5.6%)

**Table 3:** Clinical presentation of AVM stratified by grade (Low grade/High grade).

	All Patients	Low Grade	High Grade
	(N = 111)	(N = 93)	(N = 18)
AVM rupture			
Yes	59 (53.2%)	49 (5.27%)	10 (55.6%)
No	52 (46.8%)	44 (47.3%)	8 (44.4%)
Epileptic seizure			
Generalized seizure	38 (34.2%)	33 (35.5%)	5 (27.8%)
Partial seizure	14 (12.6%)	10 (10.8%)	4 (22.2%)
Neurological deficit	37 (33.3%)	28 (30.1%)	9 (50%)
Headache	54 (51.4%)	42 (45.2%)	12 (66.7%)

**Table 4:** Functional outcomes (based on mRS) by hemorrhagic presentation, AVM risk category and venous pattern drainage.

	All Patients	Good Outcome at Discharge	Poor Outcome at Discharge	Good Outcome at 9 Months	Poor Outcome at 9 Months	*p*-Value
	(N = 111)	(N = 71)	(N = 40)	(N = 90)	(N = 11)	
Ruptured AVMs						
Low grade	49	26	23	38	6	0.016
High grade	10	1	9	4	2	
Unruptured AVMs						
Low grade	44	41	3	43	1	0.001
High grade	8	3	5	5	2	
Venous drainage						
Superficial	65 (58.6%)	50	15	58	5	0.03
Deep	22 (19.8%)	11	11	17	2	
Combined	24 (21.6%)	10	14	16	4	

**Table 5:** Results of a multivariable logistic regression analysis evaluating factors associated with hemorrhagic presentation in patients with arteriovenous malformations (AVMs).

Variable	OR (Exp(B))	95% CI Lower	95% CI Upper	*p*-Value
AVM size	11.105	0.783	157.442	0.075
Side	3.818	0.278	52.505	0.316
Supplementary Martin–Spetzler score	2.429	2.429	2.429	0.016
No. of feeders	0.769	0.769	0.769	0.532
ACM feeding artery	0.375	0.026	4.845	0.439
Superficial drainage	0.111	0.017	0.709	0.020
Deep drainage	0.217	0.023	2.082	0.185
Venous ectasia	0.546	0.157	1.895	0.340
No. of drainage veins	1.589	0.775	3.254	0.206

**Table 6:** Results of a multivariable logistic regression analysis evaluating factors associated with mRS score at nine months follow-up.

Variable	OR (95% CI)	R Value	*p*-Value
Supplementary Martin–Spetzler grade	1.636 (1.265–2.115)	0.490	<0.001
AVM size	0.647 (0.453–0.925)	0.185	0.025
Side	1.000 (−0.346 to 0.363)	0.046	0.962
Deep venous drainage	1.003 (−0.308 to 0.708)	0.140	0.436
Combined venous drainage	0.866 (−0.729 to 1.452)	0.233	0.642
No. of drainage veins	1.188 (−0.059 to 3.403)	0.178	0.142
Venous ectasia present	0.880 (−0.488 to 2.231)	-	0.479
No. of feeders	1.177 (−0.074 to 2.400)	0.169	0.176
AVM grade	0.956 (−1.058 to 2.172)	0.372	0.908
Intracerebral hemorrhage	1.769 (0.072–1.070)	0.224	0.026
Intraventricular hemorrhage	2.414 (−0.403 to 5.232)	0.251	0.175

**Table 7:** Distribution of modified Rankin Scale (mRS) scores at discharge and at 9-month follow-up.

	All Patients	Low Grade	High Grade
	(N = 111, %)	(N = 93, %)	(N = 18, %)
mRS on discharge			
1	42 (37.8%)	42 (45.2%)	0
2	29 (26.1%)	25 (26.9%)	4 (22.2%)
3	11 (9.9%)	7 (7.5%)	4 (22.2%)
4	17 (15.3%)	13 (14.0%)	4 (22.2%)
5	1 (0.9%)	0	1 (5.6%)
6	11 (9.9%)	6 (6.5%)	5 (27.8%)
mRS after 9 months			
0	26 (23.4%)	26 (28.0%)	0
1	49 (44.1%)	46 (49.5%)	3 (16.7%)
2	15 (13.5%)	9 (9.7%)	6 (33.3%)
3	9 (8.1%)	6 (6.5%)	3 (16.7%)
4	1 (0.9%)	0	1 (5.6%)

**Table 8:** Perioperative complications and outcomes by risk.

	All Patients	Low Grade	High Grade	*p*-Value
	(N = 111, %)	(N = 93, %)	(N = 18, %)	
Intracerebral hemorrhage	12 (10.8%)	9 (9.7%)	3 (16.7%)	
Meningitis	15 (13.5%)	12 (12.9%)	3 (16.7%)	
Wound infection	9 (8.1%)	7 (0.5%)	2 (11.1%)	
mRS at discharge				
Good outcome	71 (64%)	67 (72%)	4 (22.2%)	0.0001
Poor outcome	40 (36%)	26 (28%)	14 (77.8%)	
mRS at 9 months				
Good outcome	90 (81.1%)	81 (87.1%)	9 (50%)	0.007
Poor outcome	10 (9.0%)	6 (6.5%)	4 (22.2%)	

**Table 9:** Correlation analysis for complications.

Correlation	Correlation Coefficient	Statistical Significance	Effect Size (Cohen’s d)
Meningitis/intracerebral hemorrhage	0.796	0.000	2.63
Meningitis/mRS	0.253	0.007	0.64
Intracerebral hemorrhage/mRS	0.305	0.001	0.52

**Table 10:** Mortality rates stratified by grade and rupture status.

	All Patients (N)	Deaths (N)	Mortality %	95% CI	*p*-Value
	111	11	9.9%	4.7–15.2%	
Low-grade AVMs	93	6	6.45%	1.5–11.4%	0.006 *
High-grade AVMs	18	5	27.8%	7.1–48.5%	
Unruptured AVMs	52	1	1.9%	0–5.6%	0.008 *
Ruptured AVMs	59	10	16.9%	7.3–26.5%	

*—represents statistically significant values.

**Table 11:** Mortality rates across AVM subgroups stratified by grade and rupture status.

	All Patients (N)	Deaths (N)	Mortality %	95% CI	*p*-Value
	111	11	9.9%		
Low-grade ubAVMs	44	0	0	4.7–15.2%	0.018 *
Low-grade rAVMs	49	6	10.1%	0–6.8%	
High-grade ubAVMs	8	1	12.5%	3.6–20.8%	0.007 *
High-grade rAVMs	10	4	40%	0–35.2%	

*—represents statistically significant values.

## Data Availability

The raw data supporting the conclusions of this article will be made available by the authors on request.

## Abbreviations

The following abbreviations are used in this manuscript:

AVM	Arteriovenous malformation
MRI	Magnetic resonance imaging
mRS	The Modified Rankin Score
ICA	Internal carotid artery
PCoA	Posterior communicating artery
ACA	Anterior cerebral artery
MCA	Middle cerebral artery
PCA	Posterior cerebellar artery
SCA	Superior cerebellar artery
PICA	Posterior inferior cerebellar artery
AICA	Anterior-inferior cerebellar artery
rAVM	Ruptured AVM
ubAVM	Unruptured AVM
